# One-year clinical outcomes following Edwards INSPIRIS RESILIA aortic valve implantation in 487 young patients with severe aortic stenosis: a single-center experience

**DOI:** 10.3389/fcvm.2023.1196447

**Published:** 2023-08-03

**Authors:** Alizee Porto, Gregoire Stolpe, Rita Badaoui, Vincent Boudouresques, Cornelia Deutsch, Cecile Amanatiou, Alberto Riberi, Vlad Gariboldi, Frédéric Collart, Alexis Theron

**Affiliations:** ^1^ Department of Cardiac Surgery, APHM, Timone Hospital, Marseille, France; ^2^ Institute for Pharmacology and Preventive Medicine, Cloppenburg, Germany

**Keywords:** aortic valve replacement, bioprosthetic valves, INPIRIS RESILIA valve, structural valve degeneration, mortality

## Abstract

**Introduction:**

The use of an aortic bioprosthesis is on the rise in younger patients with severe aortic stenosis despite the risk of accelerated structural valve degeneration (SVD). In the search for an optimal valve substitute that would not be prone to SVD, the INSPIRIS bioprosthesis represents a promising solution to lowering the risk of SVD. Here, we report the 1-year outcomes of the INSPIRIS RESILIA aortic bioprosthesis in a population of young patients who underwent aortic valve replacement.

**Methods:**

In this prospective single-center study, we included all consecutive patients receiving INSPIRIS RESILIA bioprosthesis between June 2017 and July 2021. Patients with isolated severe aortic regurgitation were excluded. Clinical assessment and transthoracic echocardiography were performed preoperatively and at 1 year post-operatively. The primary outcome was overall mortality at one year.

**Results:**

A total of 487 patients were included. The mean age was 58.2 ± 11.5 years, 75.2% were men. Most of the interventions were elective, with a mean EuroSCORE II of 4.8 ± 7.9. The valve annulus size in most cases was either 23 mm or 25 mm. Overall mortality at 1-year was 4.1%. At 1-year, 7 patients (1.4%) had a stroke, 4 patients (0.8%) had a myocardial infarction, and 20 patients (4.1%) were hospitalized for congestive heart failure. The Kaplan-Meier estimated survival rates and survival without major adverse cardiac events at 1-year were 96.4% and 96.7%, respectively. At 1-year follow-up, 10 patients (2.1%) had endocarditis and 1 patient (0.2%) had partial prosthetic thrombosis. Pacemaker implantation at 1-year post-operative was necessary in 27 patients (5.5%). Severe patient prosthesis mismatch and severe intra valvular regurgitation were 1.2% and 0.6%, respectively. The Kaplan-Meier estimated survival rates at 1-year of no infective endocarditis preoperative and infective endocarditis preoperative were 97.9 ± 0.7% and 89.5 ± 3.3%, respectively (*P* < 0.001). Excluding endocarditis-related complication, no structural valve deterioration and no valve failure requiring redo surgery were reported.

**Conclusion:**

This is the largest single-center descriptive study of the 1-year outcomes after INSPIRIS RESILIA bioprosthesis implantation. The EDWARDS INSPIRIS RESILIA bioprosthesis provides encouraging clinical outcomes with an excellent 1- year survival rates and good hemodynamic performance. Long-term studies are mandatory to assess valve durability.

## Introduction

Aortic stenosis is the most common valvular heart disease in developed countries, with a prevalence of 3.4% and an incidence of 5 in 1.000 per year among the population at age 65 years (1). Since 1960, the gold standard treatment for this disease has been surgical valve replacement. The management of these patients has changed in recent decades due to the development of the Transcatheter Aortic Valve Implantation (TAVI) technique. According to the European guidelines concerning aortic stenosis, patients over 75 years of age can benefit from TAVI (2). Conventional surgery for aortic valve replacement (AVR) is therefore currently indicated for younger patients, since it allows left ventricle mass regression by reducing LV afterload, thereby improving survival and quality of life, and avoiding the risk of congestive heart failure. The choice between a biological and a mechanical valve remains a controversial issue in younger patients, as neither type of prosthesis has shown its superiority on prognosis in patients aged between 50 and 65 years (3, 4). Moreover, both types of prosthesis have disadvantages that can impact the quality of life: bleeding risk of anti-coagulant treatments for mechanical valves and risk of structural valve degeneration (SVD) for bioprosthesis (5).

This SVD involves the unavoidable risk of reintervention, especially in patients under 65 years (6) and this management has also been modified by endovascular approach with Valve-In-Valve (VIV) aortic implantation (7).

The durability of the bioprosthesis is therefore an important issue, especially in younger patients. That is why the INSPIRIS RESILIA aortic valve with a novel model was developed by Edwards Lifesciences (Irvine, USA). It is a bioprosthetic bovine pericardial tissue valve with a special integrity preserving technology. In addition to having a phospholipid removal process, this technology allows the blockage of residual aldehyde groups known to bind calcium. Moreover, VFit technology present in this bioprosthesis was designed to allow for potential VIV procedures in the future, by increasing the size of the annular stent at the time of a new valve implantation.

Early clinical experience with the INPIRIS RESILIA valve showed encouraging results in terms of early safety and effectiveness of AVR (8, 9). Intermediate-term outcomes have shown the absence of early thrombosis events or non-calcific valve deterioration (10, 11). Five-year results have also been described, concluding in encouraging findings on the safety and hemodynamic performance of this bioprosthesis (12, 13).

However, the use of a new biological valve implies that different short and medium term studies be performed, to confirm the previous results.

Our aim was to evaluate the 1-year outcome of the INSPIRIS RESILIA aortic valve (Edwards Lifesciences) in a population of young patients who underwent AVR for severe aortic stenosis.

## Materials and methods

### Study population

In this prospective single-center study, we included all patients receiving the INSPIRIS RESILIA bioprosthesis between June 2017 and July 2021, at La Timone Hospital in Marseille, France. Patients with severe aortic stenosis were included. Patients under 18 years old, those with isolated severe aortic regurgitation, and those declining to participate in follow-up were excluded. Demographic characteristics preoperative and intraoperative variables were collected: age, sex, cardiovascular risk factors, past medical and surgical history, EuroSCORE II, and infective endocarditis (IE).

Consent was obtained from patients to participate in this anonymous publication. Our Clinical Trials registration number was RGPD2019-48.

### Surgical techniques

Since 2017, a new generation biological valve, INSPIRIS RESILIA (Edwards Lifesciences), has been utilized in our center. All patients had a full sternotomy. After completion of cardiopulmonary bypass (CPB) and cross clampage (CC), an aortotomy was performed. The choice of the type of prosthesis was left to the surgeon's discretion after valve excision and annular decalcification. AVR could be associated with concomitant surgery, including coronary artery bypass graft CABG, mitral valve repair or replacement, aortic replacement, and tricuspid valve repair or replacement.

### Follow-up

All outcomes were defined according to the Valve Academic Research Consortium-2 (VARC-2) consensus document (14).

At 30-days, early complications were overall and cardiovascular mortality, major bleeding (defined by re-exploration for bleeding or need for >4 blood unit replacement), renal failure, respiratory failure, sternal infection, stroke, cardiogenic shock, cardiac tamponade and pacemaker implantation.

One-year outcomes included overall and cardiovascular mortality, disabling stroke, myocardial infarction, rehospitalization for congestive heart failure (CHF), pacemaker implantation and IE.

### Transthoracic echocardiography follow-up

Hemodynamic follow-up was assessed by echocardiography analyzed by a senior cardiologist.

Left ventricular ejection fraction (LVEF) was estimated using the Simpson Biplane method, and the Devereux formula was used to calculate LV mass index. The Continuous Doppler technique was used to estimate the transaortic gradient. The effective orifice area (EOA) was calculated using the continuity equation, and the indexed effective orifice area (iEOA) was calculated by dividing the EOA by body surface area. Moderate Patient Prosthesis Mismatch (PPM) was defined by an iEOA between 0.85 and 0.65 cm/m^2^ or between 0.75 and 0.55 cm/m^2^ if body mass index > 30 kg/m^2^, and severe PPM by an iEOA below 0.65 cm/m^2^ or 0.55 cm/m^2^ if body mass index > 30 kg/m^2^. Trans Valvular Regurgitation (TVR) and Para Valvular Leak (PVL) were described according to classification as mild, moderate or severe.

### Endpoints

The primary endpoint was overall mortality at 1 year.

Secondary endpoints were cardiovascular mortality, major adverse cardiac events (defined by rehospitalization for congestive heart failure, stroke or myocardial infarction), early complications and at 1-year follow-up, evolution of functional status New York Heart Association (NYHA), hemodynamic follow-up, and mortality-morbidity of IE.

An analysis was carried out in three groups, according to the age of the patient: 50 years old or younger, between 50 and 65 years old, and 65 years or older. Preoperative data and follow-up were compared between these groups.

Another subgroup analysis was performed according to the IE preoperative status.

### Statistical analysis

Statistical analyses were performed for the total study population on available data. Categorical variables are reported as absolute numbers and percentages (%). Continuous variables are presented as mean ± standard deviation (SD) or median [interquartile range (IQR)]. All continuous variables were checked for distribution using the Kolmogorov-Smirnov test. Comparisons were made using a Pearson's Chi-Square or Fisher's exact test for categorical variables. For continuous variables comparisons were performed according to their distribution, using a Student's *t*-test or Mann-Whitney_*U* test for 2-group comparison. Comparisons between more than 2 groups were made using the Kruskal-Wallis-H test, including a paired comparison if there was seen a significant difference between subgroups. Wilcoxon signed rank test for paired data was used for comparing echo data between baseline and follow-up. Kaplan-Meier estimates are provided for survival and safety outcomes. A *P*-value of <0.05 was considered statistically significant. All statistical analyses were performed using IBM SPSS Statistics version 29 (IBM, Armonk, New York).

## Results

**Figure 1:**
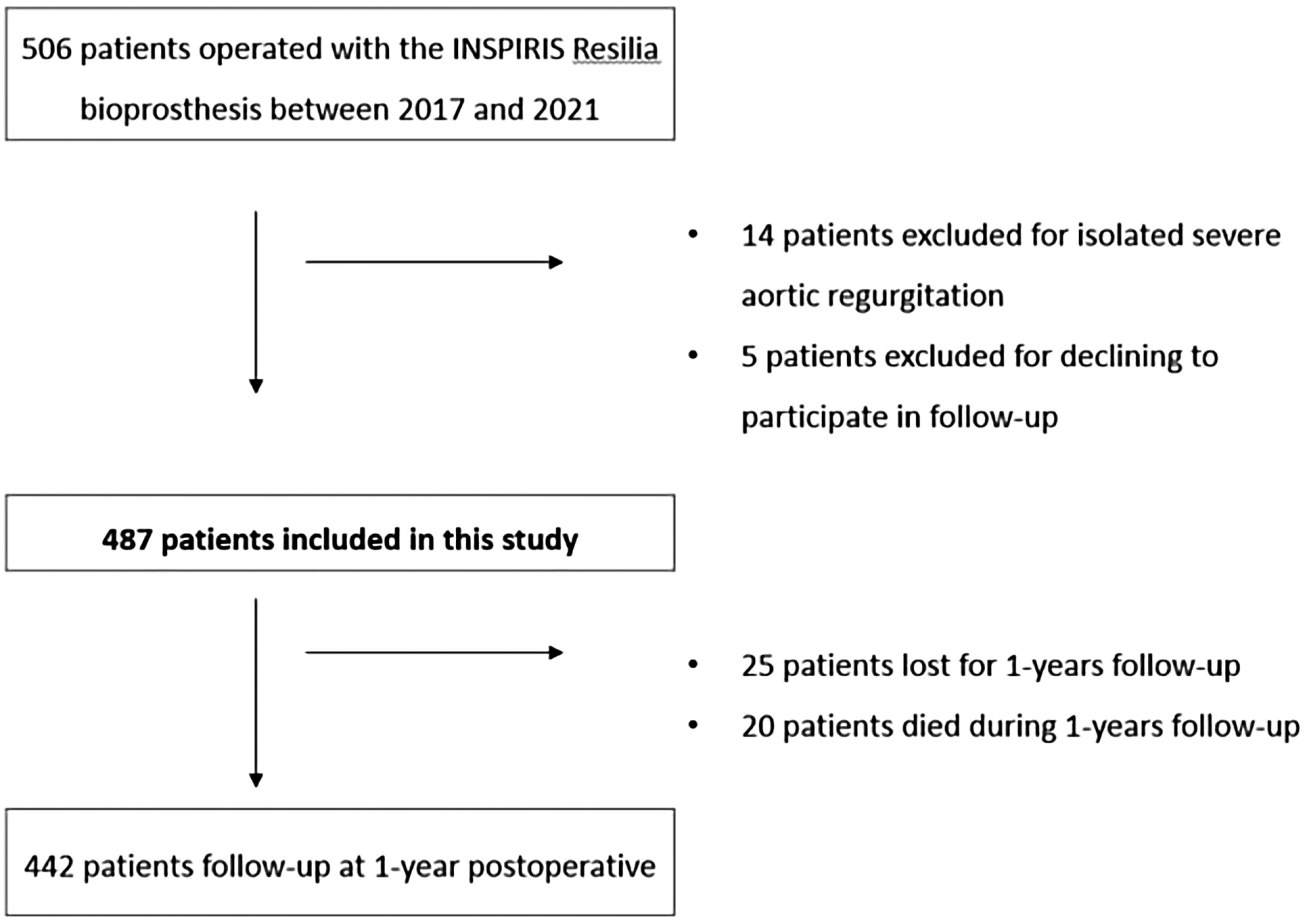
Patient disposition flowchart.

Between June 2017 and July 2021, 506 patients were treated for AVR with an INSPIRIS RESILIA bioprosthesis. Fourteen patients were excluded for isolated aortic regurgitation and five patients declined to participate in this study. In total, 487 patients were included in the study (Figure 1).

### Baseline and procedural data

The mean patient age was 58.2 ± 11.5 years, 21.1% were under 50 years-old, with male predominance (75.2%). EuroScore II was 4.8 ± 7.9 and 227 patients (46.6%) were in NHYA Class III or IV. Most of the interventions were elective (363 patients, 74.5%). Ninety-one patients (18.7%) had endocarditis, 72 patients (14.8%) were redo surgery and 152 patients (31.2%) had aortic stenosis without aortic regurgitation. Baseline characteristics including cardiovascular risk factor are presented in Table 1.

**Table 1:** Demographics characteristics among 489 patients treated with bioprosthetic INSPIRIS aortic valve for severe aortic stenosis. Comparison according to the age of patient.

Variable	Overall *n* = 487 (100%)	Age ≤ 50 years *n* = 103 (21.1%)	Age 51–64 years *n* = 216 (44.4%)	Age ≥ 65 years *n* = 168 (34.5%)	*P* value
Age, mean ± SD (range)	58.2 ± 11.5 (17–78)	40.5 ± 9.0	58.5 ± 4.0	68.6 ± 3.0	*–*
Male, *n* (%)	366 (75.2)	77 (74.8)	172 (79.6)	117 (69.6)	0.08
EuroSCORE II, mean ± SD (range)	4.8 ± 7.9 (0.5–58.4)	5.3 ± 9.6	4.2 ± 7.2	5.2 ± 7.7	0.008*.
Body mass index, mean ± SD (range)	26.3 ± 4.9 (15.8–53)	25.0 ± 4.8	26.8 ± 5.2	26.4 ± 4.5	0.002*
Syncope, *n* (%)	18 (3.7)	6 (5.8)	11 (5.1)	1 (0.6)	0.01
Nyha I–II, *n* (%)	260 (53.4)	57 (55.3)	111 (51.4)	92 (54.8)	0.08
Nyha III–IV, *n* (%)	227 (46.6)	46 (44.7)	105 (48.6)	76 (45.2)	0.08
IE preoperative, *n* (%)	91 (18.7)	30 (29.1)	40 (18.5)	21 (12.5)	0.003
Hypertension, *n* (%)	240 (49.3)	22 (21.4)	117 (54.2)	101 (60.1)	<0.001
Hyperlipidemia, *n* (%)	165 (33.9)	7 (6.8)	81 (37.5)	77 (45.8)	<0.001
Diabetes insulin treated, *n* (%)	33 (6.8)	1 (0.9)	20 (9.3)	12 (7.1)	0.02
Peripheral vascular disease, *n* (%)	23 (4.7)	1 (0.9)	11 (5.1)	11 (6.5)	0.09
Myocardial infarction, *n* (%)	13 (2.7)	1 (0.9)	10 (4.6)	2 (1.2)	0.08
Liver disease, *n* (%)	6 (1.2)	2 (1.9)	3 (1.4)	1 (0.6)	0.58
Pulmonary disease, *n* (%)	31 (6.4)	5 (4.9)	11 (5.1)	15 (8.9)	0.28
Renal insufficiency, *n* (%)	23 (4.7)	2 (1.9)	8 (3.7)	13 (7.7)	0.08
Smoker, *n* (%)	240 (49.3)	51 (49.5)	125 (57.9)	64 (38.1)	<0.001
History of cancer, *n* (%)	60 (12.3)	6 (5.8)	25 (11.6)	29 (17.3)	0.02
Surgery redo, *n* (%)	72 (14.8)	21 (20.4)	36 (16.7)	15 (8.9)	0.02

IE, infective endocarditis; NYHA, New York heart association; SD, standard derivation.

Continuous variables are mean ± SD (range). Categorical variables are reported as absolute numbers and percentages. *P*-value calculated using Chi-Square test.

*Comparison for non-parametric continuous values using Kruskal-Wallis-H. Comparison for three groups according to the age: ≤50 years-old, 50–65 years-old, ≥65 years-old. A *P*-value of <0.05 was considered statistically significant.

**Figure 2:**
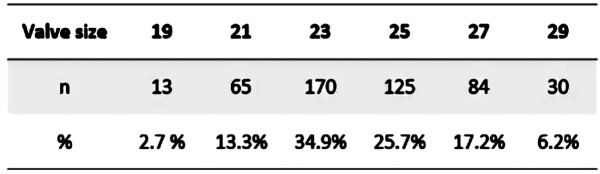
INSPIRIS RESILIA valve distribution according to size.

Perioperatively, 210 (43.1%) patients had a concomitant procedure performed of which 114 (23.4%) had aortic replacement, 45 (9.2%) had mitral valve surgery replacement or repair and 36 (7.4%) had CABG. The mean CC and CPB times were 85.9 ± 33.9 min and 112.6 ± 49.5 min, respectively. INSPIRIS valves sizes 23 mm (170 patients, 34.9%) and 25 mm (125 patients, 25.7%) were the most implanted (Figure 2).

### Early mortality and morbidity

Mean duration of hospitalization was 16.6 ± 12.6 days.

At 30-days follow-up, 8 patients (1.6%) had died: 5 (1.0%) following cardiogenic shock, 2 (0.4%) following multiorgan failure, and 1 (0.2%) following mesenteric ischemia. Early complications occurred in 66 patients (13.6%) and included respiratory failure in 19 patients (3.9%), acute renal failure requiring dialysis in 12 patients (2.5%), cardiogenic shock necessitating extra-corporeal membrane oxygenation in 8 patients (1.6%), stroke in 4 patients (0.8%), cardiac tamponade in 12 patients (2.5%), major bleeding in 28 patients (5.7%), 16 (3.3%) of whom needed reintervention, and sternal infection in 4 (0.8%). Pacemaker implantation was required in 23 patients (4.7%) at discharge.

### One-year outcomes

**Figure 3:**
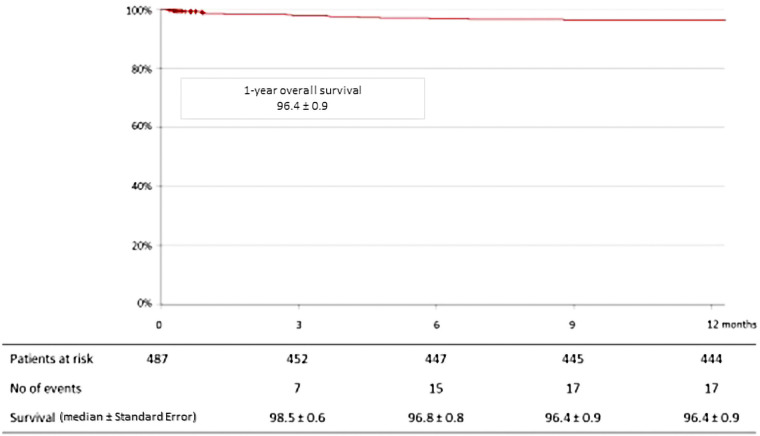
Kaplan-Meier curve for overall survival at 1-year among 489 patients treated with bioprosthetic INSPIRIS RESILIA aortic valve for severe aortic stenosis.

**Figure 4:**
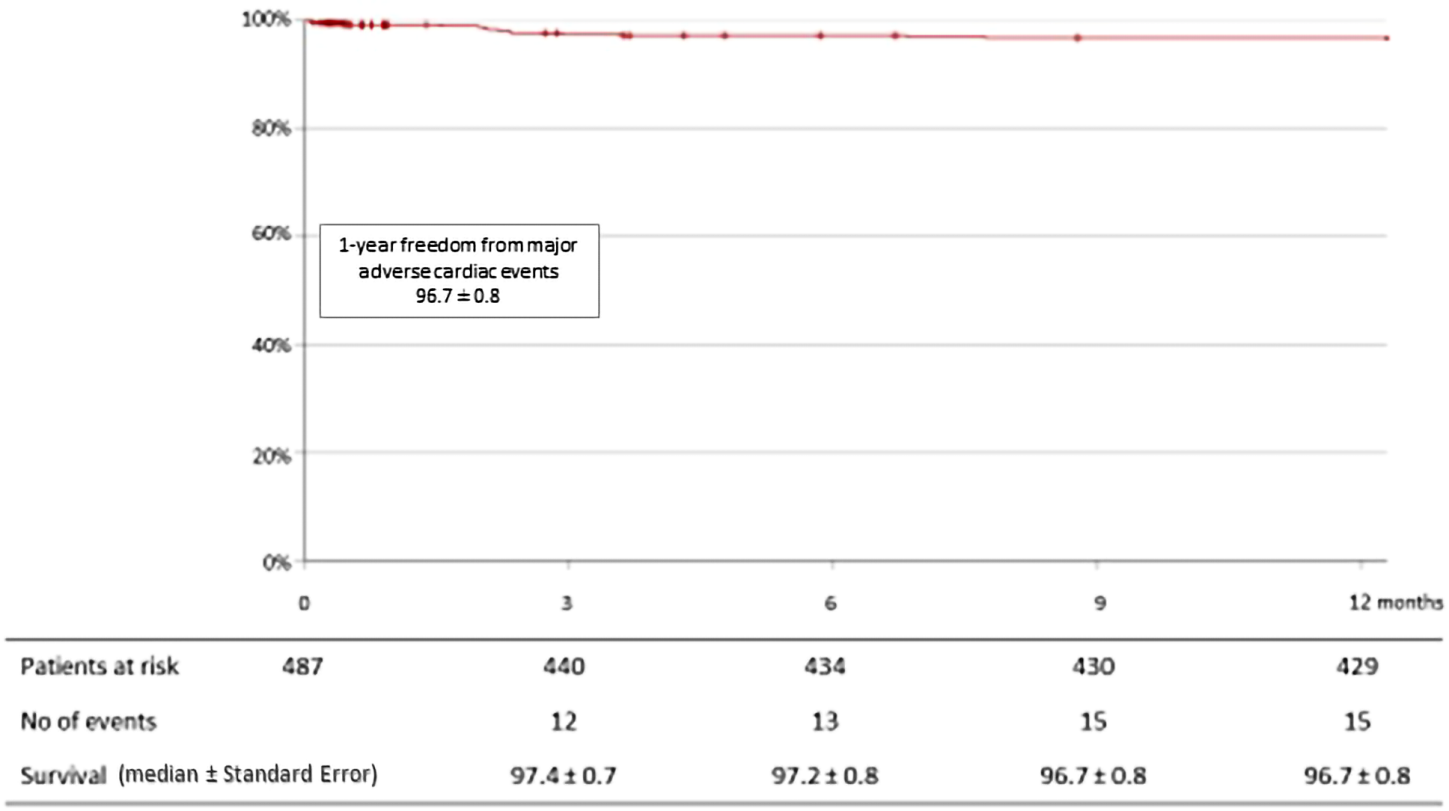
Kaplan-Meier curve for freedom from major adverse cardiac events (stroke, myocardial infarction and hospitalization for congestive heart failure) at 1-year among 489 patients treated with bioprosthetic INSPIRIS RESILIA aortic valve for severe aortic stenosis.

At 1-year post implant, 25 (5.1%) patients were lost to follow-up;

  12 death occurred: 4 (0.8%) following congestive heart failure, 2 (0.4%) following COVID-19 infections, 1 (0.2%) following gastrointestinal bleeding, 2 (0.4%) following cerebral hemorrhages, and 1 (0.2%) from indeterminate causes. In total, the mortality rate at 1-year was 4.3% (20 patients). The Kaplan-Meier estimated survival rate at 1-year was 96.4 ± 0.9% (Figure 3). Regarding complications at 1-year, 7 patients (1.4%) had a stroke, 4 patients (0.8%) had myocardial infarction and 20 patients (4.1%) were hospitalized for congestive heart failure. The Kaplan-Meier estimated survival without major advance cardiac events at 1-year was 96.7 ± 0.8% (Figure 4).

Pacemaker implantation for new-onset conduction disorders at 1-year was required in 27 patients (5.5%).

Ten patients (2.3%) developed IE at 1-year follow-up, 4 of which had recurrence IE.

**Figure 5:**
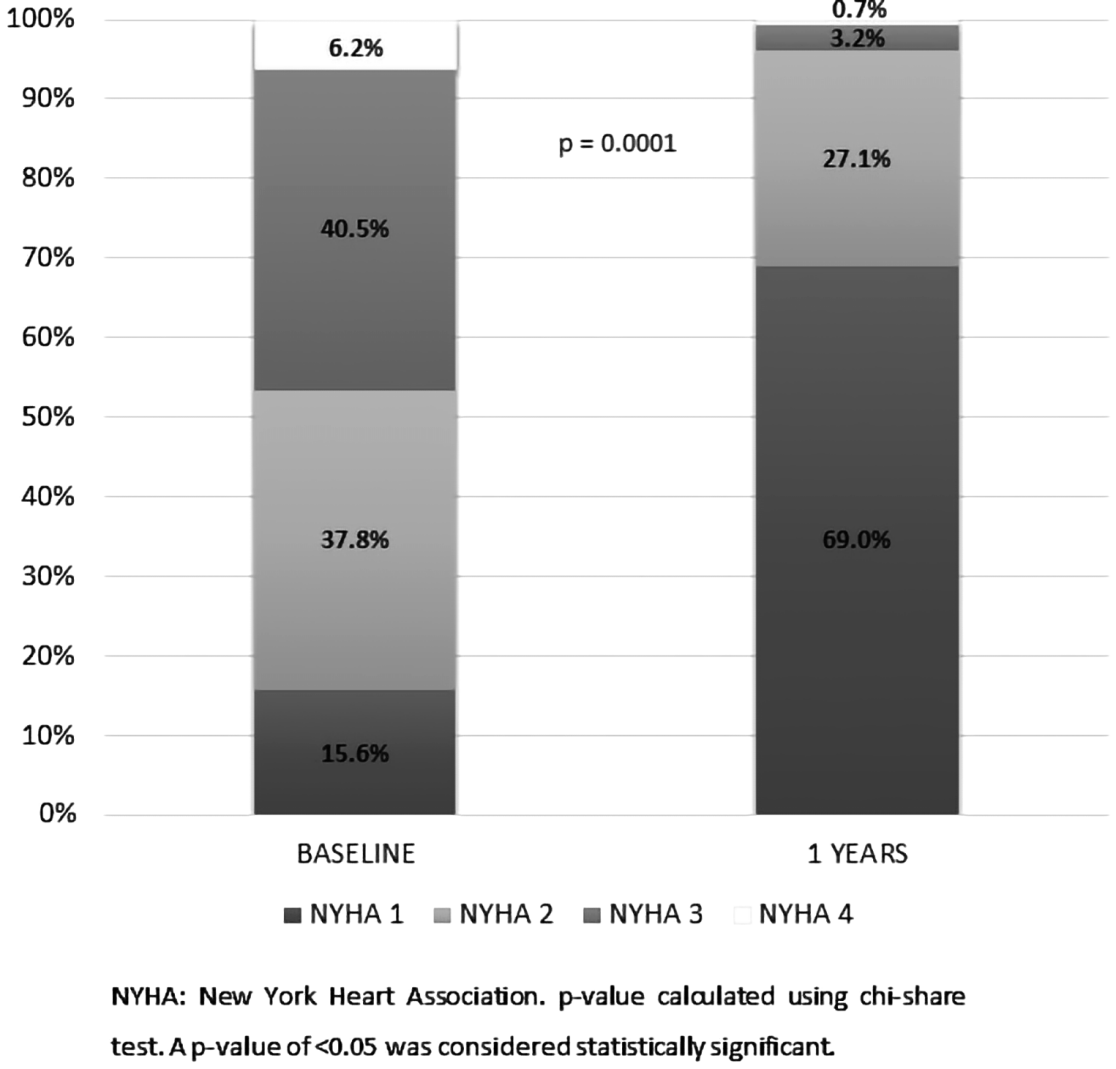
Change in New York heart association status from baseline to 1-year follow-up among 489 patients treated with bioprosthetic INSPIRIS RESILIA aortic valve for severe aortic stenosis. NYHA, New York heart association. *P*-value calculated using Chi-Square test. A *P*-value of <0.05 was considered statistically significant.

Functional status was significantly improved (Figure 5): 227(46.7%) patients had NYHA III/IV preoperatively whereas 17 patients (3.9%) had NYHA III/IV postoperatively (*P* < 0.05).

### Hemodynamic follow-up

At 30-days, any implant malposition was reported, and 6.2% (30 patients) with moderate PPM and 1.4% (7 patients) with severe PPM were observed.

Concerning the evolution of the hemodynamic parameters at 1-year, the mean gradient decreased from 49 mmHg (43;55) preoperatively to 9 mmHg (7;12) at 1-year post-operative (*P* < 0.001), and the peak velocity decreased from 4.4 m/s (4.0;4.9) to 2.1 m/s (1.8;2.4) (*P* < 0.001) (Table 2).

**Table 2:** Transthoracic echocardiography characteristics from preoperative to 1-year post-operatively among 489 patients treated with a bioprosthetic INSPIRIS RESILIA aortic valve for severe aortic stenosis. Comparison according to the age of patient.

	Preoperative, median (IQR)	1-year postoperative, median (IQR)					
	*n* = 487	Overall *n* = 442	Age ≤ 50 *n* = 93	Age 51–64 *n* = 198	Age ≥ 65 *n* = 151	*P* value^†^	*P* value*
LVEF (%)	60 (50;65)	60 (55;66)	62 (55;66)	60 (54.5;65)	65 (60;69.7)	**<0**.**001**	0.005
Aortic valve area index (cm^2^/m^2^)	0.4 (0.4;0.5)	1.0 (0.8;1.3)	0.9 (0.8;1.2)	1.0 (0.8;1.2)	1.1 (0.9;1.3)	**<0**.**001**	0.2
Peak velocity (m/s)	4.4 (4.0;4.9)	2.1 (1.8;2.4)	2.0 (1.7;2.3)	2.2 (1.8;2.5)	2.1 (1.8;2.3)	**<0**.**001**	0.5
Mean transvalvular gradient (mmHg)	49 (43;55)	9 (7;12)	10 (8;13)	9 (7;12)	9 (7;12)	**<0**.**001**	0.2
Stroke volume index (ml/m^2^)	45 (38.5;53.2)	44 (38;51.1)	41 (33;52)	43 (38;49.1)	47.5 (43;60.5)	**0**.**495**	0.08

LVEF, left ventricular ejection fraction; IQR, interquartile range.

Data are median (IQR).

^†^*P*-value calculated using Wilcoxon test for paired samples, comparison preoperative and 1-year follow-up.

*Comparison for non-parametric continuous values using Kruskal-Wallis-H, comparison for three groups according of the age: ≤50 years-old, 50–65 years-old, ≥65 years-old. A *P*-value of <0.05 was considered statistically significant.

Moderate and severe PPM were observed in 51 (10.5%) patients and 6 (1.2%) patients, respectively. Concerning prosthetic regurgitation, moderate and severe TVR were reported in 2 patients (0.4%) and in 1 patient (0.2%), respectively. Moderate or severe PVL was observed in 7 patients (1.4%) and 3 patients (0.6%), respectively.

One patient (0.2%) developed partial valvular thrombosis with favorable evolution under anticoagulant treatment.

### Clinical outcomes according to age

Three subgroups were analyzed: 103 patients (21.1%) were 50 years old or younger, 216 (44.4%) were between 50 and 65 year old and 168 (34.5%) were65 years orolder. Patients under 50 years-old had a higher EuroSCORE II (5.3 ± 9.6) and presented more often with preoperative IE (29.1%); 20.4% of the cases were redo surgeries. Patients between 50 and 65 years old were predominantly male (79.6%), had hypertension (54.2%) and were smokers (57.9%) (Table 1).

During the follow-up, overall mortality was 6.6%, 3.4% and 4.4% for the three successive age groups (*P* = 0.53). Moreover, there was no significant difference in term of morbidity according to major adverse cardiac events (Table 3).

**Table 3:** At 1-year post-operatively, mortality and morbidity among 489 patients treated with bioprosthetic INSPIRIS aortic valve for severe aortic stenosis. Comparison according to the age of patient.

Event at 1-year, *n* (%)	Overall *n* = 487 (100%)	Age ≤ 50 years *n* = 103 (21.1%)	Age 51–64 years *n* = 216 (44.4%)	Age ≥ 65 years *n* = 168 (34.5%)	*P* value
Overall Mortality	20 (4.)	6 (6.1)	7 (3.4)	7 (4.4)	0.53
Cardiovascular mortality	9 (1.9)	3 (3.0)	4 (2.0)	2 (1.3)	0.56
Stroke	7 (1.6)	0 (0.0)	3 (1.5)	4 (2.7)	0.29
Myocardial infarction	4 (0.9)	1 (1.1)	3 (1.5)	0 (0.0)	0.35
Hospitalisation for CHF	9 (2.0)	3 (3.2)	5 (2.5)	1 (0.7)	0.29
Major adverse cardiac events	20 (4.5)	4 (4.3)	11 (5.6)	5 (3.3)	0.67
New permanent pacemaker	27 (5.5)	5 (4.9)	11 (5.1)	11 (6.6)	0.78
IE postoperative	10 (2.3)	3 (3.2)	5 (2.5)	2 (1.3)	0.60

CHF, congestive heart failure; IE, infective endocarditis.

Categorical variables are reported as absolute numbers and percentages. *P*-value calculated using Chi-Square test, comparison for 3 groups according of the age: ≤50 years-old, 50–65 years-old, ≥65 years-old. A *P*-value of <0.05 was considered statistically significant.

Concerning transthoracic echocardiography characteristics at 1-year, the group of patients 65 years or older had a greater increase in LVEF (65%, *P* = 0.005) and in stroke volume index (47.5 ml/m^2^, *P* = 0.08) compared with the other two groups. Evolution of the aortic valve area index, the peak velocity and the mean trans-valvular gradient showed no difference between these three groups (Table 2).

### Infective endocarditis

Patients with preoperative IE were younger (median age 54.7 ± 12.7 years, *P* = 0.002), with a higher EuroSCORE II (11.5 ± 12.4, *P* < 0.001) compared to the IE-free group (median age 59.0 ± 11.0 and EuroSCORE II 3.3 ± 5.5). Elective procedure was realized in 12.1% of the cases (11 patients) and redo surgery in 38.5% (35 patients), with a significant difference in the IE-free group, 88.9% (352 patients, *P* < 0.001) and 9.3% (37 patients, *P* < 0.001), respectively.

**Figure 6:**
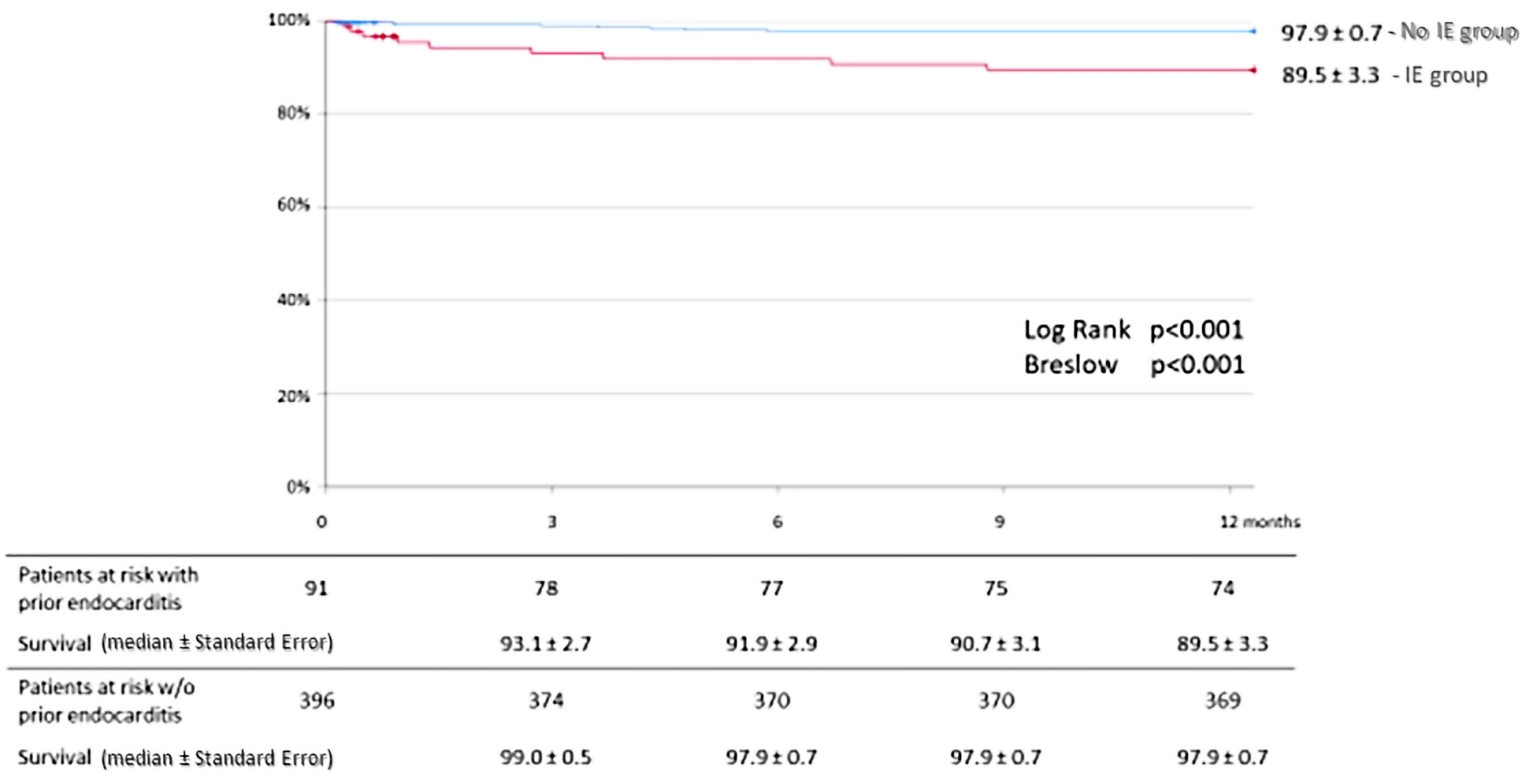
Kaplan-Meier curves for overall survival at 1-year in sub-group analysis of patients with and without prior infective endocarditis. IE, infective endocarditis.

The Kaplan-Meier estimated survival rates at 1-year for IE-free group and IE group were 97.9 ± 0.7% and 89.5 ± 3.3%, respectively (*P* < 0.001) (Figure 6). Major adverse cardiac event accounted for 4.4% (16 patients) in IE-free group and 5.3% (4 patients) in the IE group.

Concerning prosthetic regurgitation, one severe TVL was reported in the IE group (Table 4).

**Table 4:** Postoperative mortality and morbidity at 1-year follow-up according to endocarditis preoperative status among 489 patients treated with bioprosthetic INSPIRIS aortic valve for severe aortic stenosis.

Event at 1-year, *n* (%)	No IE *n* = 396 (81.3%)	IE *n* = 91 (18.7%)	*P* value
Overall mortality	11 (2.9)	9 (10.7)	<0.01
Cardiovascular mortality	4 (1.0)	5 (5.9)	0.01
Stroke	7 (1.9)	0 (0.0)	0.5
Myocardial infarction	3 (0.8)	1 (1.3)	1.0
Hospitalisation for CHF	6 (1.6)	3 (4.0)	0.4
Major adverse cardiac event	16 (4.4)	4 (5.3)	0.8
IE postoperative	6 (1.6)	4 (5.3)	0.1
New permanent pacemaker	12 (3.0)	15 (16.5)	<0.01
Severe PPM	6 (1.5)	0 (0.0)	0.5
Severe transaortic regurgitation	0 (0.0)	1 (1.3)	0.2
Severe paravalvular leak	1 (1.3)	2 (2.2)	0.2

CHF, congestive heart failure; IE, infective endocarditis; PPM, patient prosthesis mismatch. Categorical variables are reported as absolute numbers and percentages. *P*-value calculated using Chi-Square test. A *P*-value of <0.05 was considered statistically significant.

Redo cardiac surgery at 1-year was required in 6 patients (1.2%): 3 patients had recurrence IE and 3 patients had IE post-operative.

Excluding IE related causes, no structural valve deterioration and valve failure requiring redo surgery were described.

## Discussion

This prospective study offers results on almost 500 patients undergoing AVR with the INSPIRIS RESILIA Edwards bioprosthesis. One-year overall mortality was 4.1% and cardiovascular mortality was 1.9%. A low rate of major cardiac events was observed, as well as satisfactory functional and hemodynamic outcomes. The rate of moderate or severe TVR (0.6%) and the rate of moderate or severe PVL (2.1%) were low. Excluding IE related causes, no structural valve deterioration and valve failure requiring redo surgery were described.

Short and medium term results for the INSPIRIS RESILIA Edwards bioprosthesis have been described in previous studies: low morbidity and mortality, along with a significant decrease in symptoms (10, 11, 15, 16).

In our study, there was no SVD described at 1-year and the risk of valvular reintervention was only related to infective IE. The causes of SVD are threefold, including chemical mechanisms, hemodynamic rheological stresses and an inappropriate immunological response by the host. Regarding the chemical mechanisms, residual phospholipid and aldehyde moieties in processed bioprosthetic valve leaflets are important contributors to the formation of calcium phosphate crystals (17, 18). Glutaraldehyde, an element used during the manufacturing of the bioprosthesis to fix and preserve the biological tissue, seems to preserve the valve's properties of resistance and elasticity and diminishes its antigenicity. However, it paradoxically promotes degeneration by increasing cellular permeability and the process of valvular calcification (19).

To address this problem, the RESILIA tissue of the Edwards INSPIRIS RESILIA bioprosthesis was developed. Due to the neutralization of the free aldehydes present on the prosthetic valve tissue by an industrial chemical process and the conservation of the prosthesis in a glutaraldehyde-free environment, it is likely that the structural degeneration of the prosthetic calcium is slowed down *in vivo*.

The COMMENCE Trial (20) included 689 patients and reported interesting findings at 5-year follow-up. Freedom from all-cause mortality, all-cause reoperation, and study valve explant were 89.2%, 98.7%, and 99.0%, respectively. Freedom from valve thrombosis, non-SVD, and SVD were all 100%.

Currently, there is no international consensus regarding the management of patients with aortic stenosis who are under 65 years old. In our study, 1 in 5 patients were under 50 years and almost half of the patients were between 50 and 65 years. The American guidelines recommend considering the replacement of the aortic valve by a bioprosthesis starting from the age of 50 (21), while the more conservative European guidelines advocate the use of bioprosthesis in patients over 60 years old (2). The American guidelines reasoning is based on the improved durability of the bioprosthesis which, and especially in the advent of aortic VIV procedure, avoids a surgical re-intervention. Therefore, it is important to consider the longterm cost and impact of this procedure on a young population. Improving the longevity of bioprosthetic valves will make it possible to optimize a patient's lifetime management.

On the other hand, VIV TAVI has emerged as a less invasive alternative to redo surgery for high and intermediate-risk patients. In a meta-analysis, Bruno et al. reported that 30-day all-cause and cardiovascular mortality were significantly lower with VIV compared with redo surgery, with risk of major bleeding significantly lower in patients undergoing VIV (22). However, Nalluri et al. (23) reported no significant difference in 30-day mortality and 1-year mortality between VIV TAVI and redo surgical AVR. Regarding hemodynamic efficiency, Gozdek et al. (7) considered that redo AVR offered superior echocardiographic outcomes with lower incidence of patient-prosthesis mismatch, fewer paravalvular leaks and lower mean postoperative aortic valve gradients. Surgical redo should remain the standard of care, particularly in the low-risk population. The VIV TAVI offers an effective, less invasive alternative for high- risk patients and those who are contraindicated for surgery. In our study, redo surgery was less frequent in patients over 65 years-old and conventional surgery was preferred in younger patients.

INSPIRIS RESILIA valve with VFit technology was developed by adding one sliding zoneto the metal frame of the stent to facilitate the expansion of the prosthetic ring during VIV procedures. The objective is to allow for the implantation of a larger diameter prosthesisthus reducing the risk of mismatch. Moreover, this avoids “cracking” the outcomes of which appear unfavorable with a higher risk of in-hospital mortality and major bleeding, as well as modest improvements in hemodynamic status (24).

We also analyzed IE which affected almost 1 in 5 preoperative patients in our study. At 1-year, the mortality rate of these patients was 10.7%. This rate seems low compared to literature data (25), and can be explained by the younger patient population benefiting from an INSPIRIS RESILIA bioprosthesis and the absence of data regarding annular involvement.

In our study, post-operative IE was responsible for valvular degeneration and for a higher risk of reintervention at 1-year follow-up. Prosthetic valve IE is the most severe form of IE, the incidence was 0.3%–1.2% per patient-years and the actual risk of recurrence of IE varies between 2 and 6% (25).

This study has limitations. First, this is a single arm study without comparative groups.

Moreover we only studied follow up for 1-year. Consequently, we cannot fully address the question of long term durability. The generalizability of the results may be limited by the absence of a predetermined sample size and post-hoc power analysis. Concerning patients under 50 years old, the data is difficult to interpret because of the preoperative overrepresentation of IE in this population. This explains the excess of adverse event in this population. Concerning IE, our data seem to favor INSPIRIS use in this population, however the low number of patients in the study is insufficient to fully address this question. The fact of not excluding patients treated for endocarditis is a bias of confusion, with more complicated consequences. However, this allows us to have a real-work practice.

## Conclusion

The Management Of Aortic Stenosis In Young Subjects remains an important topic of discussion. The new genera­tion INPIRIS RESILIA aortic valve bioprosthesis demonstrated excellent hemodynamic performance and safety outcomes at one-year follow-up. Long-term studies are needed to further assess valve durability.

## Data Availability

The raw data supporting the conclusions of this article will be made available by the authors, without undue reservation.
